# Simulated cardiopulmonary bypass: a high fidelity model for developing and accessing clinical perfusion skills

**DOI:** 10.1186/s41077-023-00269-w

**Published:** 2024-01-02

**Authors:** Bruce E. Searles, Jeffrey B. Riley, Edward M. Darling, Jason R. Wiles

**Affiliations:** 1 Department of Cardiovascular Perfusion, College of Health Professions, SUNY Upstate Medical University, 750 E. Adams St, Syracuse, NY 13210 USA; 2 Departments of Biology and Science Teaching, College of Arts and Science, Syracuse University, Syracuse, USA

**Keywords:** Cardiopulmonary bypass, Clinical perfusionist, Cardiac surgery, Education, Simulation, Validation, Fidelity

## Abstract

**Background:**

Traditionally, novice perfusionists learn and practice clinical skills, during live surgical procedures. The profession’s accrediting body is directing schools to implement simulated cardiopulmonary bypass (CPB) into the curriculum. Unfortunately, no CPB simulation models have been validated. Here we describe the design and application of a CPB simulation model.

**Methods:**

A CPB patient simulator was integrated into a representative operative theater and interfaced with a simple manikin, a heart-lung machine (HLM), clinical perfusion circuitry, and equipment. Participants completed a simulation scenario designed to represent a typical CPB procedure before completing an exit survey to assess the fidelity and validity of the experience. Questions were scored using a 5-point Likert scale.

**Results:**

Participants (*n* = 81) contributed 953 opinions on 40 questions. The participants reported that the model of simulated CPB (1) realistically presented both the physiologic and technical parameters seen during CPB (*n* = 347, mean 4.37, SD 0.86), (2) accurately represented the psychological constructs and cognitive mechanisms of the clinical CPB (*n* = 139, mean 4.24, SD 1.08), (3) requires real clinical skills and reproduces realistic surgical case progression (*n* = 167, mean 4.38, SD 0.86), and (4) would be effective for teaching, practicing, and assessing the fundamental skills of CPB (*n* = 300, mean 4.54, SD 0.9). Participants agreed that their performance in the simulation scenario accurately predicted their performance in a real clinical setting (*n* = 43, mean 4.07, SD 1.03)

**Conclusion:**

This novel simulation model of CPB reproduces the salient aspects of clinical CPB and may be useful for teaching, practicing, and assessing fundamental skills.

**Supplementary Information:**

The online version contains supplementary material available at 10.1186/s41077-023-00269-w.

## Introduction

During open heart surgery procedures that require cardiopulmonary bypass (CPB), it is common to arrest the patient’s heart to allow the surgeon to operate on a motionless and bloodless heart. During this time of cardiac arrest, the patient’s life is dependent on the skilled actions made by a clinical perfusionist operating a heart and lung machine (HLM).

Historically, perfusionist trainees had to practice their fundamental skills during real cardiac surgical procedures. Given the gravity of the role of the perfusionist, there is great interest in including simulation early in the curriculum [1, 2]. Several commercial CPB simulators have been marketed [3–5], and educational programs are applying simulation for training and assessment with increasing frequency [6–14]. Our educational program has been developing an innovative simulation model of CPB for use in the training of clinical perfusionists. Towards this, we completed a national survey to identify the fundamental skills conducted by clinical perfusionists during the operation of the heart and lung machine (HLM) [15], and the normal limits of physiologic and technical parameters that clinical perfusionists manage [16]. These metrics have been used to validate the benchtop performance of a commercially distributed patient simulator designed to be a patient surrogate for simulated CPB procedures [17].

However, in order to confidently apply this innovative technology to develop and assess students’ pre-clinical perfusion skills, the simulation model (which includes the simulator technology within the context of the physical environment and the presentation of the scenario) must demonstrate technical and psychological fidelity. Additionally, evidence of content and predictive validity is a prerequisite for applying the model to skill assessment and determination of a student’s readiness for live clinical preceptorship and advancement through an academic skills curriculum.

**Fig. 1:**
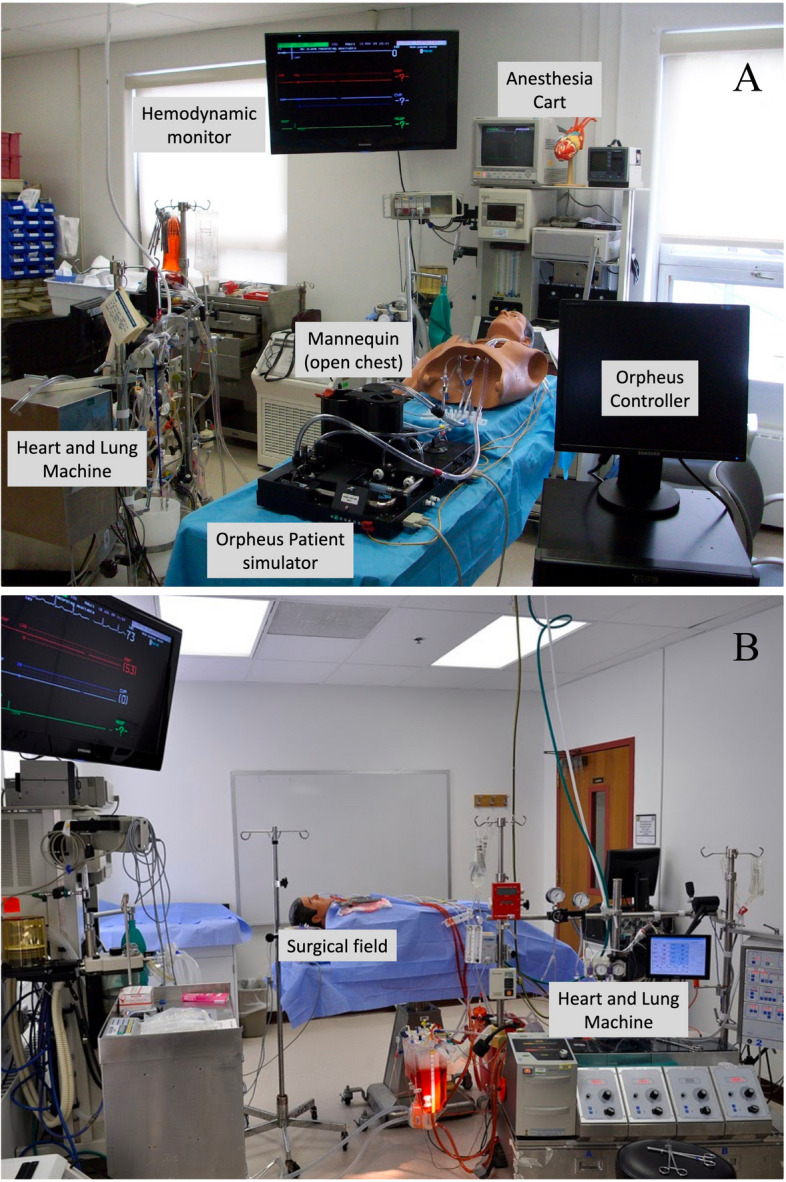
The CPB simulation suite. **A** Arrangement of the clinical and simulation equipment in the CPB simulation suite. The Orpheus^TM^ patient simulator is positioned on an operating table and interfaced with a manikin torso. The simulator’s controller is located on the patient’s left side for easy access by the facilitator who plays the role of the surgeon in the scenario. The participant/perfusionist operates the perfusion equipment including the HLM on the patient’s right side. The anesthesia cart is at the patient’s head and the hemodynamic monitor screens are located above the anesthesia cart. **B** Image of the surgical field from the point-of-view of the participant/perfusionist

Through the use of exit surveys completed by simulation subjects, the authors aimed to specifically measure the validity and fidelity of our CPB model in four specific categories: (1) physiological and technical fidelity, (2) psychological fidelity and believability, (3) content and predictive validity, and (4) relevance and didactic usefulness Fig. 1.

## Methods

### The simulation suite

The simulation suite is in an academic building at a medical university. A commercially manufactured  cardiopulmonary bypass (CPB) simulator (Orpheus^TM^) was interfaced with the upper torso of a simple, hollow manikin to create an interactive manekin model of a cardiac surgery patient with a median sternotomy and vascular instrumentation for hemodynamic monitoring and cannulation. The Orpheus^TM^ Perfusion Simulator is a computer-controlled, hydro-mechanical task-trainer technology which represents a patient’s circulatory system and incorporates computerized physiologic and pharmacodynamic algorithms that simulate the hemodynamic response of a patient receiving CPB [4]. The simulator and manikin were placed on an operating table and draped as a patient receiving cardiac surgery. The manikin was interfaced with an anesthesia machine, hemodynamic monitoring systems, and a heart lung machine (HLM). The HLM was equipped with clinical extracorporeal circuitry. In this configuration, the HLM’s blood pumps, monitoring systems, and safety devices were fully functional, creating a clinically relevant three-way interface between the perfusionist, the HLM, and the simulated patient. An additional movie file shows this in more detail, see the Additional file “CBP Simulation (Demo)” in the online repository (https://osf.io/fe75k).

In this model, a minimum of two participants are necessary to conduct a scenario. The facilitator is responsible both for the technical operation of the simulator along with playing an embedded actor who helps guide the simulation scenario as the cardiac surgeon would direct a clinical procedure. The participant is the subject of the simulation scenario.

### Procedure and simulation scenario

#### Phase 1: orientation

Participants were oriented to the simulation suite by an experienced facilitator and given a minimum of 1 h to acquaint themselves with the equipment and the layout of the monitoring systems within the room.

#### Phase 2: practice session

Participants were assisted by the simulation facilitator and were led through a practice session allowing them to operate the equipment on vignettes of fundamental CPB skills.

#### Phase 3: simulation scenario 3

Participants completed a clinically relevant pre-bypass checklist [18], and the Standardized CPB case simulation was conducted. The progression of the simulation scenario was based on typical surgical techniques and included the fundamental skills and the normal clinical parameters associated with the operation of the HLM [15, 16].

#### Phase 4 Post-simulation exit survey and debriefing

We have applied this perfusion skills simulation model to the presentation and development of fundamental and crisis management CPB skills for more than a decade. We have surveyed the participants about their experience as a metric of our internal quality assurance program and these surveys were analyzed for this study.

In phases 1–3 in order to maximize the believability of the experience, the simulation suite was treated “as-if” it were an actual operating room. Participants entered the room wearing scrubs, facemasks, caps, and gloves, interacted with the clinical equipment with clinically acceptable behaviors, and when the facilitator was in character as the surgeon/actor all communication was modeled after typical operating room interactions.

### Participants and groups

Survey results were sorted into two groups based on the live clinical experience level of the participants as follows:

- *Experienced* group had previously performed greater than 20 clinical procedures
- *Inexperienced group* had previously performed fewer than 20 clinical procedures.

No educational treatment, method, or experience was applied to either group for this study. Data is initially presented as experienced and inexperienced groups to investigate the range of applicability for different learner groups, and to justify pooling the data from both groups for analysis and before making conclusions.

Each participant performed a standardized simulation scenario that emphasized the clinical perfusionist’s role in cardiopulmonary bypass procedures. The progress of the scenario was led by an experienced facilitator with training and experience in clinical instruction of perfusion students and simulation facilitation. Following their simulation scenario, the participants completed an exit survey and were debriefed.

### Data collection questionnaires

Our internal quality control exit survey program was reviewed by our Institutional Review Board (IRB) and was determined to be exempt from ongoing review. Participants were surveyed for their demographics and they evaluated statements using a 5-point Likert scale to ascertain how well the simulation experience reproduced a clinical procedure. Four very similar versions of the survey instruments were used over the period of data collection. The 5-point scale used to evaluate the questions was the same for all versions of the survey (1 = strongly disagree, 2 = disagree, 3 = neutral, 4 = agree, 5 = strongly agree). There was a total of 40 questions. A compendium of the questions can be found in the Supplemental material-Addendum located in an online repository; https://osf.io/yd3qa/files/osfstorage/64b6e5fcf29691025bf0797f.

To aggregate the responses from different versions of the survey instrument into a single data pool, the questions from each version were assigned to categories according to their content. The questions supported the post hoc development of the following four categories:

1. Category I: physiological and technical fidelity (18 questions)

This category assessed the realism of the equipment and how the simulation scenario represented patient variables such as hemodynamic blood pressures and blood gas parameters as well as technical skills such as initiation of CPB, administration of cardioplegia solutions, and weaning from the HLM.

2- Category II: psychological fidelity and believability (5 questions)

This category assessed whether the simulation accurately represented the psychological constructs and cognitive mechanisms of the clinical CPB and if the scenario accurately represented the non-technical skills (e.g., communication) used during CPB procedures.

3- Category III: content and predictive validity (5 questions)

This category assessed content validity by asking whether the simulation completely and accurately incorporated the perfusionist’s skills and abilities which are included in the same clinical scenario. Predictive validity was assessed by questions asking if the subjects believed that their performance in the simulated environment predicts their performance in the real clinical environment under similar circumstances.

4- Category IV: relevance and didactic usefulness (12 questions)

This category assessed the relevance and didactic usefulness of this model by asking questions about its utility for teaching, practicing, and assessing the fundamental skills of CPB.

### Data analysis

Likert scores for each question were analyzed. The scores were not normally distributed when tested with the Kolmogorov–Smirnov test. The mean (M), standard deviation (SD), median (m), and interquartile range (IQR) are reported. Analysis of variance (ANOVA) between Likert means was used for comparisons between groups for each question and category.

## Results

### Demographics of participants

Between 2009 and 2018, 81 individuals (39 inexperienced groups, 42 experienced groups) were surveyed. The inexperienced group consisted of students from our own educational program (intramural perfusion students *n* = 19) and students from other accredited perfusion education programs (extramural perfusion students *n* = 20). The experienced group consisted of extramural perfusion students (*n* = 26) and practicing clinical perfusionists (expert *n* = 16). Student participants had performed 0–50 proctored clinical cases as part of their educational program. Expert perfusionist participants had independently performed more than 2,000 clinical cases after their training (M 2253 SD 1.871, m 2000 IQR 2000). The complete demographic dataset is available as a table online in a Supplemental material-Addendum: https://osf.io/n8m2g.

The summary results for each of the 4 categories are presented in Table 1. The individual results for each of the 40 questions are available online in a Supplemental material-Addendum: https://osf.io/yd3qa/files/osfstorage/64b6e5fcf29691025bf0797f.

**Table 1:** Participants summary results for all categories

	Experienced group			Inexperienced group			Between groups	Category total		
Response category	*n*	M (SD)	m (IQR)	*n*	M (SD)	m (IQR)	*p* value	*n*	M (SD)	m (IQR)
Category I Physiologic and technical fidelity (18 questions)	192	4.44 (0.72)	5 (1)	155	4.27 (0.99)	5 (1)	0.070	347	4.37 (0.86)	5 (1)
Category II Psychological fidelity and believability (5 questions)	71	4.34 (0.93)	5 (1)	68	4.09 (1.23)	4.5 (1)	0.181	139	4.24 (1.08)	5 (1)
Category III Content and predictive validity (5 questions)	93	4.29 (0.90)	5 (1)	74	4.47 (0.82)	5 (1)	0.186	167	4.38 (0.86)	5 (1)
Category IV Relevance, Didactic, and Usefulness (12 questions)	149	4.57 (0.80)	5 (1)	151	4.50 (0.99)	5 (1)	0.483	300	4.54 (0.90)	5 (1)
Grand total	505	4.44 (0.81)	5 (1)	448	4.35 (1.00)	5 (1)	0.24	953	4.41 (0.91)	5 (1)

Legend: *n* is the number of responses to all questions in each category on post-simulation surveys. *M* = mean, *SD* = standard deviation, *m* = median, *IQR* = interquartile range

Between groups *p* value is ANOVA test between experienced (> 20 human cases) versus Inexperienced means

The participants *agreed* or *strongly agreed* that the scenario realistically presented both the physiologic and technical parameters seen during CPB (Category I: aggregate of 18 questions, *n* = 347, M 4.37 SD 0.86; m 5, IQR 1). This category asked about the fidelity of simulated patient parameters (*n* = 43), the haptics of the CPB equipment (*n* = 81), the realism of procedures such as cannula placement (*n* = 9), initiation of CPB (*n* = 38), patient hemodynamic management (*n* = 46), patient arterial blood gas (ABG) management (*n* = 54), cardioplegia delivery (*n* = 38), and weaning from CPB (*n* = 38). The participants agreed or strongly agreed that the scenario accurately represented the psychological constructs and cognitive mechanisms of the clinical CPB (Category II: aggregate of 5 questions, *n* = 139, M 4.24 SD 1.08; m 5, IQR 1). This category asked about the psychological fidelity experienced by the participants during the scenario including the believability of the scripted dialogue (*n* = 29). This category also asked if the experience felt like a real cardiac surgery (*n* = 43) and if the fidelity of the scenario generated nervous feelings in the participants (*n* = 24). In this category, participants *strongly agreed* that the simulation suite was a safe learning environment (Category II question 1: *n* = 43, M 4.88 SD 0.32; m 5 IQR 0).

Overall, participants agreed or strongly agreed that the scenario completely and accurately incorporates the perfusionist’s skills and abilities (Category III: aggregate of 5 questions, *n* = 167, M 4.38 SD 0.86; m 5, IQR 1) and that their performance in the simulated environment predicts their performance in the real clinical environment under similar circumstances While on 39 of the 40 questions, there was no difference between the opinions of the experienced and the inexperienced groups, there was a significant difference (*p* = 0.007) in opinions between the groups regarding the predictive validity of their performance in the scenario. Inexperienced participants *strongly agreed* or *agreed* that their performance in the scenario was an accurate representation of how they would perform in the same situation in a real perfusion case (Category III question 5: *n* = 18, M 4.51 SD 0.51; m 5 IQR 1) while the experienced group *agreed* or were *neutral* about the same statement (Category III question 5: *n* = 25, M 3.72 SD 1.17; m 4 IQR 2). Participants agreed or strongly agreed that the simulation model would be effective for teaching, practicing, and assessing the fundamental skills of CPB (Category IV: aggregate of 12 questions, *n* = 300, M 4.54 SD 0.9; m 5, IQR 1). This category asked about the participants’ opinions on the relevance, didactic content, and usefulness of the simulation model including the effectiveness of the prebriefing (*n* = 123) and debriefing (*n* = 71). Additionally, participants commented on the model’s effectiveness as a method for practicing skills (*n* = 29) and ranked the value of the experience relative to a clinical observation in the operating room or a classroom lecture (*n* = 41).

## Discussion

In the USA, the accreditation of educational programs for preparing entry-level clinical perfusionists is directed by the Committee for the Accreditation of Allied Health Education Programs (CAAHEP) and its Co-Accrediting partner the Accreditation Committee for Perfusion Education (ACPE). These governing bodies represent the collective voice of the cardiac surgery community through the representation of 7 professional organizations in the field of Cardiac Surgery, Cardiac Anesthesia, and Cardiovascular Perfusion. The accrediting body’s most recent update to the Standards and Guidelines for the Accreditation of Educational Programs in Perfusion (section III.C) [1, 2] requires educational programs to include simulated clinical scenarios in the curriculum and recommends the use of high-fidelity simulation. We present here the first known report on the validity and fidelity of a high-fidelity simulation model of CPB. This evidence is a necessary prerequisite to the implementation and incorporation of high-fidelity simulation into the national curriculum. We have demonstrated an innovative simulation model which could be reproduced in any educational program to meet the accreditation standards for the profession. The participants in this study covered a wide range of experience levels from students to seasoned clinicians and they agreed or strongly agreed that this simulation model represents the same clinical skills that are taught in educational programs and are used for patient care and that their performance in the simulation scenario predicts how they would perform in the same situation in a real clinical perfusion case. Furthermore, the participants strongly agreed that this model represents an effective way to practice clinical perfusion skills and that this simulation model is more effective than lecture or clinical observation.

The realistic, high-fidelity, presentation of the clinical perfusion environment requires more than a technically validated patient simulator. Our group has previously reported the results of a large national study which identified a professional consensus of skills and parameters which are very commonly applied to clinical perfusion [15, 16] and demonstrated the operational limits of a commercially marketed perfusion simulator device which reproduce the physiological and technical variables that mimic real clinical procedures [17]. This study advances this body of work by demonstrating that these validated elements can be presented within the context of a simulated clinical scenario and create a high-fidelity learning environment that may be realistic enough to augment learning by developing transferable skills in a physically and psychologically safe student-focused learning space. Historically, perfusion education programs applied “wet labs” in which the students practiced skills while “pumping a bucket”. With the development of computerized patient simulators our profession's educational bucket has become much more technically savvy and automated. However, to be more than a fancy wet lab, CPB patient simulators still require the support of a simulation theater and the purposeful presentation of a well-designed scenario in a realistic environment. The model presented here applies the *as-if* concept and helps the participant to suspend their disbelief and enter into the scenario [19] by providing a reasonable level of physical reality elements and framing them within the context of semantic modulations that help the participant to engage in the alternate reality of the simulation scenario. Physically, this simulation suite is obviously not a real operating room and the hard plastic manikin would not be confused with a real patient. However, the perfusion equipment, which is a separate technology from the patient simulator, is all real clinical equipment. The HLM, CPB circuit, clamps, transducers, heater-coolers, are all actual clinical devices and perfectly reproduce the look and haptics of a clinical perfusionist’s machine interface. Furthermore, the inclusion of a live actor in the role of a surgeon, communicating in a clinically relevant manner to direct the scenario brings realistic non-technical elements into the overall scenario and adds to the fidelity of the experience.

Our innovative high-fidelity simulation model has the potential to enhance student learning and protect patients from the student's predictable learning curve. Incorporating this model into the national curriculum may accelerate the trainees' development of a transferable skill in an environment that is student-focused and without introducing avoidable learning curve risks into the care of real patients [20, 21]. This model could be used in a pre-clinical skill development curriculum which includes deliberate practice [22] of deconstructed skills [23, 24], and encourages trainees to learn from their mistakes [25] on fundamental skills [15] as well as low-volume-high-risk events [26]. This curriculum could be supported with the strategic use of video capture [27] so that the trainees could review their own performance as well as the performances of their peers.

The model validated herein provides a means by which pressing questions in the field may be approached. The application of simulation for the initial training and continuing competency assessment of clinical perfusionist's skills is an under-researched subject. There are no published simulation curricula, assessment rubrics, or training scenarios for educators to reference. These materials are needed for the instruction and evaluation of students and the continuing education and competency assessment of experienced practicing clinicians. Additionally, we must investigate if the techniques and technologies of perfusion simulation have an impact on skill development or maintenance as compared to the traditional Halsted model of skill development and regular clinical practice.

If fully implemented in the national curriculum, as recommended in the standards [1, 2], high-fidelity simulation of CPB has the potential to enhance entry-level education and patient care by developing students' pre-clinical skills prior to their clinical preceptorships where their actions and their learning curve are part of a patient’s medical care.

## Conclusion

Our key findings in this survey report are that this simulated CPB model (1) realistically reproduces valid technical and physiologic parameters, (2) feels real with participant agreement on its psychological fidelity and believability, (3) demonstrates content validity with agreement that it accurately incorporates the perfusionist’s skills and abilities, (4) has predictive validity as there was no disagreement that performance was an accurate representation of how they would perform in the same situation in a real perfusion case.

Competent performance of CPB involves a complex synthesis of cognitive, psychomotor, and affective learning domains. Our model appears to create a safe learning environment and captures the level of fidelity, believability, and validity needed for teaching, practicing, and assessing fundamental skills of cardiopulmonary bypass.

## Limitations

The post-session survey questions became more focused over the study period. This necessitated the joining of multiple related survey items into categories which captured the entire data set and facilitated analysis of all of the participant responses.

As with the statistical analysis of any Likert scale ordinal data, we assumed the Likert numbers were related to each other with measurable intervals which is a requirement for ANOVA.

In Memoriam: Jeff Riley 1950–2021; Perfusionist, Teacher, Friend.

### Supplementary Information

The datasets supporting the conclusions of this article are available in the Center for Open Science repository, https://osf.io/yd3qa/?view_only=eeed1d035f6c4dcfbff541f9dbee7e35.

**Additional file 1.** CPB Simulation (DEMO).

**Additional file 2.** OSM Demographics.

**Additional file 3.** OSM every question.

## Data Availability

All relevant data have been submitted. The datasets generated and/or analyzed during the current study are available in the Open Science Framework repository, https://osf.io/yd3qa/?view_only=eeed1d035f6c4dcfbff541f9dbee7e35.

## Abbreviations

CPB: Cardiopulmonary bypass
HLM: Heart-lung machine
IQR: Interquartile range
M: Mean
m: Median
SD: Standard deviation
ANOVA: Analysis of variance
*n*: Number of participant answers

## References

[1] Accreditation Committee for Perfusion Education. Standards and Guidelines for the Accreditation of Educational Programs in Perfusion. 2018. Cited 2021 Apr 2. Available from: http://caahep.org/documents/file/News-And-Announcements/PerfusionStandards2012.pdf.

[2] Fernandez A (2010). Simulation in perfusion: where do we go from here?. Perfusion..

[3] Nesbitt JC, Michaud NM, Brakebill A, Deppen SA, Williams P (2018). Month-long cardiac surgery boot camp: A proposal to jumpstart resident training. J Thorac Cardiovasc Surg..

[4] Morris RW, Pybus DA (2008). “Orpheus” cardiopulmonary bypass simulation system. J Extra Corpor Technol..

[5] Raasch D, Austin J, Tallman R (2010). Self-priming hemodynamic reservoir and inline flow meter for a cardiopulmonary bypass simulation. J Extra Corpor Technol..

[6] Sistino JJ, Michaud NM, Sievert AN, Shackelford AG (2011). Incorporating high fidelity simulation into perfusion education. Perfusion..

[7] Austin JW, Riley JB (2005). Principles of simulation applied to perfusion technology: 2005 update. J Extra Corpor Technol..

[8] Austin JW, Casidy B, Olson T, Trahan A, Bowers M, Calkins (2002). Transferring air force flight simulation training effectiveness to university-based cardiopulmonary bypass simulation training: “a model for success”. J Extra Corpor Technol..

[9] Lansdowne W, Machin D, Grant DJ (2012). Development of the orpheus perfusion simulator for use in high-fidelity extracorporeal membrane oxygenation simulation. J Extra Corpor Technol..

[10] Turkmen A, Rosinski D, Noyes N (2007). A simulator for perfusion training. Perfusion..

[11] Ninomiya S, Tokaji M, Tokumine A, Kurosaki T (2009). Virtual patient simulator for the perfusion resource management drill. J Extra Corpor Technol..

[12] Ninomiya S, Tokumine A, Yasuda T, Tomizawa Y (2007). Development of an educational simulator system, ECCSIM-Lite, for the acquisition of basic perfusion techniques and evaluation. J Artif Organs..

[13] Tokaji M, Ninomiya S, Kurosaki T, Orihashi K, Sueda T (2012). An educational training simulator for advanced perfusion techniques using a high-fidelity virtual patient model. Artif Organs..

[14] Gierig S, Merkle F, Pawelke C, Müller-Plath G (2020). Simulation in perfusion: evaluating the efficacy of a specific training with eye-tracking. Perfusion..

[15] Searles B, Darling E, Riley J, Wiles JR (2019). Fundamental clinical skills of adult cardiopulmonary bypass: results of the 2017 national survey. Perfusion..

[16] Searles B, Darling EM, Riley JB, Wiles JR (2020). Survey of the routine practice limits for physiologic and technical parameters managed by clinical perfusionists during adult cardiopulmonary bypass. J Extra Corpor Technol..

[17] Searles BE, Darling EM, Riley JB, McNich J, Ruffa E, Wiles JR. Objective content validation of the hemodynamic and technical parameters of the OrpheusTM Cardiopulmonary Bypass Simulator. J Extra Corpor Technol. 2021;53(4):263–69. 10.1182/ject-53-263. 10.1182/ject-53-263 PMC8717731 34992316

[18] American Society of Extracorporeral Technolgy. Perfusion Checklist. 2004. Cited 2021 Apr 2. Available from: https://www.amsect.org/page/perfusion-checklist.

[19] Muckler VC (2017). Exploring suspension of disbelief during simulation-based learning. Clin Simul Nurs.

[20] Gallagher AG, Hart M, Cleary D, Hamilton C, McGlinchey K, Kiely P (2020). Proficiency based progression simulation training significantly reduces utility strikes; a prospective, randomized and blinded study. PLoS one.

[21] Seymour NE, Gallagher AG, Roman SA, O’Brien MK, Bansal VK, Andersen DK (2002). Virtual reality training improves operating room performance: results of a randomized, double-blinded study. Ann Surg..

[22] Rowse PG, Dearani JA (2019). Deliberate practice and the emerging roles of simulation in thoracic surgery. Thorac Surg Clin.

[23] Giacomino K, Caliesch R, Sattelmayer KM (2020). The effectiveness of the Peyton’s 4-step teaching approach on skill acquisition of procedures in health professions education: a systematic review and meta-analysis with integrated meta-regression. PeerJ.

[24] Krautter M, Weyrich P, Schultz J-H, Buss SJ, Maatouk I, Jünger J (2011). Effects of Peyton’s four-step approach on objective performance measures in technical skills training: a controlled trial. Teach Learn Med.

[25] Millwood S. Developing a platform for learning from mistakes: changing the culture of patient safety amongst junior doctors. BMJ Qual Improve Rep. 2014;3(1):u203658.w2114. 10.1136/bmjquality.u203658.w2114. 10.1136/bmjquality.u203658.w2114 PMC4949613 27493733

[26] Charrière J-M, Pélissié J, Verd C, Léger P, Pouard P, de Riberolles C (2007). Survey: retrospective survey of monitoring/safety devices and incidents of cardiopulmonary bypass for cardiac surgery in France. J Extra Corpor Technol.

[27] Mazer L, Varban O, Montgomery JR, Awad MM, Schulman A (2022). Video is better: why aren’t we using it? A mixed-methods study of the barriers to routine procedural video recording and case review. Surg Endosc..

