# Supplementary material for: Simulated cardiopulmonary bypass: a high fidelity model for developing and accessing clinical perfusion skills
Source: Adv Simul (Lond). 2024 Jan 2;9:1. doi: 10.1186/s41077-023-00269-w (PMC10763050; doi:10.1186/s41077-023-00269-w)
Supplement: Supplementary file 2 — Additional file 2. OSM Demographics. [file 41077_2023_269_MOESM2_ESM.pdf]

OSM Appendix - Demographics: Participant caseloads by years and experience

		Experienced		Inexperienced	
**Subject Groups**	**Year**	**Count**	**Cases M (SD), m (IQR)**	**Count**	**Cases M (SD), m (IQR)**
**Intramural**	2009	0		3	12 (8), 10 (0)
	2010	0		0	
	2012	0		7	3 (2), 3 (4)
	2018	0		9	1 (2), 0 (3)
**Extramural**	2009	5	76 (43), 88 (82)	8	7 (8), 2 (14)
	2010	3	87 (98), 30	3	7 (12)
	2012	0		0	
	2018	18	41 (11), 42 (20)	9	2 (3), 0 (5.5)
**Expert**	2009	0		0	
	2010	0		0	
	2012	9	878 (874), 450 (1400)	0	
	2018	7	3186 (2011), 3000 (3800)	0	
	**Total**	42	752 (1437), 60 (584)	39	4 (6), 2 (5)

Legend: Intramural subjects are students in the authors educational program. Extramural subjects are students at other accredited educational programs. Expert subjects are practicing clinical perfusionists. Year represents the 4 versions of the survey. Count is the number subjects completing the survey. M = Mean, SD = Standard Deviation, m = median, IQR = Interquartile Range. Between Groups p Value is ANOVA test between Experienced (> 20 human cases) versus Inexperienced means.
