# Supplementary material for: Simulated cardiopulmonary bypass: a high fidelity model for developing and accessing clinical perfusion skills
Source: Adv Simul (Lond). 2024 Jan 2;9:1. doi: 10.1186/s41077-023-00269-w (PMC10763050; doi:10.1186/s41077-023-00269-w)
Supplement: Supplementary file 3 — Additional file 3. OSM every question. [file 41077_2023_269_MOESM3_ESM.htm]

|  |  |  |  |  |  |  |  |  |  |  |
| --- | --- | --- | --- | --- | --- | --- | --- | --- | --- | --- |
| **Addendum 1** | | | | | | | | | | |
| **Category: Survey Questions** | **Experienced** | | | **Inexperienced** | | | **p Value** | **Total** | | |
|  | **n** | **Mean (SD)** | **Med (IQR)** | **n** | **Mean (SD)** | **Med (IQR)** | **Between Experience** | **n** | **Mean (SD)** | **Med (IQR)** |
| **Category 1: Opinions on physiologic and technical fidelity** | | | | | | | | | | |
| Physiologic fidelity: The simulated patient parameters behaved like a live human surgical patient | 25 | 4.20 (0.76) | 4 (1) | 18 | 4.50 (0.62) | 5 (1) | 0.233 | 43 | 4.33 (0.72) | 4 (1) |
| This simulation experience realistically recreated (from the perfusionist�s perspective): The CPB simulator equipment is very realistic | 9 | 4.67 (0.50) | 5 (1) | 0 | \* | \* | \* | 9 | 4.67 (0.50) | 5 (1) |
| The CPB simulator equipment is very realistic | 9 | 4.56 (0.53) | 5 (1) | 20 | 4.45 (1.00) | 5 (1) | 0.769 | 29 | 4.48 (0.87) | 5 (1) |
| Technical fidelity: The equipment feels like real CPB equipment | 25 | 4.04 (1.10) | 4 (1) | 18 | 4.44 (0.70) | 5 (1) | 0.227 | 43 | 4.21 (0.97) | 4 (1) |
| This simulation experience realistically recreated (from the perfusionist�s perspective): Cannula placement verification and testing | 9 | 4.67 (0.50) | 5 (1) | 0 | \* | \* | \* | 9 | 4.67 (0.50) | 5 (1) |
| This simulation experience realistically recreated (from the perfusionist�s perspective): CPB initiation and good bypass assessment | 9 | 4.67 (0.50) | 5 (1) | 0 | \* | \* | \* | 9 | 4.67 (0.50) | 5 (1) |
| Initiation of CPB was realistic | 10 | 4.70 (0.48) | 5 (1) | 19 | 4.40 (1.01) | 5 (1) | 0.422 | 29 | 4.52 (0.87) | 5 (1) |
| This simulation experience realistically recreated (from the perfusionists perspective):� Partial versus full CPB | 9 | 4.56 (0.53) | 5 (1) | 0 | \* | \* | \* | 9 | 4.56 (0.53) | 5 (1) |
| This simulation experience realistically recreated (from the perfusionist�s perspective): Weaning and termination from CPB | 9 | 4.78 (0.41) | 5 (0.5) | 0 | \* | \* | \* | 9 | 4.78 (0.41) | 5 (0.5) |
| Weaning and termination were realistic | 9 | 4.56 (0.53) | 5 (1) | 20 | 4.10 (1.21) | 4.5 (1) | 0.291 | 29 | 4.24 (1.06) | 5 (1) |
| The simulation experience realistically recreated (from the perfusionist�s perspective): Cardioplegia delivery | 9 | 4.67 (0.25) | 5 (1) | 0 | \* | \* | \* | 9 | 4.67 (0.25) | 5 (1) |
| Cardioplegia delivery was realistic | 9 | 4.56 (0.53) | 5 (1) | 20 | 4.40 (1.05) | 5 (1) | 0.678 | 29 | 4.45 (0.91) | 5 (1) |
| The simulation experience realistically recreated (from the perfusionist�s perspective): Management of CPB parameters: SvO2 | 8 | 4.63 (0.52) | 5 (1) | 0 | \* | \* | \* | 8 | 4.63 (0.52) | 5 (1) |
| This simulation experience realistically recreated (from the perfusionist�s� perspective): Management of CPB parameters: PO2/PCO2 | 8 | 4.63 (0.27) | 5 (1) | 0 | \* | \* | \* | 8 | 4.63 (0.27) | 5 (1) |
| This simulation experience realistically recreated (from the perfusionist�s perspective): The patient variables (ABPs and ABGs) are very realistic | 9 | 4.56 (0.53) | 5 (1) | 0 | \* | \* | \* | 9 | 4.56 (0.53) | 5 (1) |
| This simulation experience realistically recreated (from the perfusionist perspective): Management of CPB parameters b. Hemodynamics | 8 | 4.38 (0.52) | 4 (1) | 0 | \* | \* | \* | 8 | 4.38 (0.52) | 4 (1) |
| Patient hemodynamics were realistic | 9 | 4.33 (0.50) | 4 (1) | 20 | 4.00 (1.12) | 4 (1) | 0.405 | 29 | 4.10 (0.98) | 4 (1) |
| Physiologic Fidelity: The simulated patient parameters behaved like a live human surgical patient: ABG management was realistic | 9 | 4.00 (1.32) | 5 (3) | 20 | 4.00 (1.03) | 4 (1) | 0.999 | 29 | 4.00 (1.10) | 4 (2) |
| **Category 1: Opinions on physiologic and technical fidelity totals** | **192** | **4.44 (0.72)** | **5 (1)** | **155** | **4.27 (0.99)** | **5 (1)** | **0.070** | **347** | **4.37 (0.86)** | **5 (1)** |
|  | | | | | | | | | | |
| **Category 2: Opinions on psychological fidelity and believability** | | | | | | | | | | |
| The simulation suite was a safe learning environment for me | 25 | 4.84 (0.37) | 5 (0) | 18 | 4.94 (0.24) | 5 (0) | 0.332 | 43 | 4.88 (0.32) | 5 (0) |
| Psychological fidelity: I felt like I was participating in a real cardiac surgical procedure | 25 | 4.00 (1.04) | 4 (2) | 18 | 4.44 (0.62) | 4 (1) | 0.152 | 43 | 4.19 (0.91) | 4 (1) |
| My interactions with the surgeon and the anesthesiologist were realistic | 9 | 4.56 (0.53) | 5 (1) | 20 | 4.00 (1.21) | 4 (2) | 0.202 | 29 | 4.17 (1.07) | 4 (1) |
| The simulation experience realistically recreated (from the perfusionist�s perspective): I was nervous when using the equipment | 9 | 4.22 (0.97) | 4 (1) | 0 | \* | \* | \* | 9 | 4.22 (0.97) | 4 (1) |
| I was nervous when using the equipment | 3 | 3.00 (1.73) | 4 (1) | 12 | 2.58 (1.38) | 2.5 (3) | 0.661 | 15 | 2.67 (1.95) | 3 (3) |
| **Category 2: Opinions on psychological fidelity and believability totals** | **71** | **4.34 (0.93)** | **5 (1)** | **68** | **4.09 (1.23)** | **4.5 (1)** | **0.181** | **139** | **4.24 (1.08)** | **5 (1)** |
|  | | | | | | | | | | |
| **Category 3: Opinions on content and predictive validity** | | | | | | | | | | |
| Content validity: The skills I used in the scenario are the same skills I use in real perfusion cases | 25 | 4.64 (0.64) | 5 (1) | 18 | 4.61 (0.61) | 5 (1) | 0.795 | 43 | 4.63 (0.62) | 5 (1) |
| The information / skills covered today have been taught in the curriculum | 9 | 4.89 (0.33) | 5 (0) | 20 | 4.25 (1.21) | 5 (1) | 0.134 | 29 | 4.45 (1.06) | 5 (1) |
| The scenario represented a real-life situation | 25 | 4.28 (0.74) | 4 (1) | 18 | 4.61 (0.61) | 5(1) | 0.164 | 43 | 4.42 (0.70) | 5 (1) |
| This simulation experience realistically recreated (from the perfusionists perspective): The surgical progression | 9 | 4.22 (0.67) | 4 (1) | 0 | \* | \* | \* | 9 | 4.22 (0.67) | 4 (1) |
| Predictive validity - My performance in his scenario is an accurate representation of how I would perform in the same situation in a real perfusion case | 25 | 3.72 (1.17) | 4 (2) | 18 | 4.56 (0.51) | 5 (1) | 0.011 | 43 | 4.07 (1.03) | 4 (1) |
| **Category 3: Opinions on psychological fidelity and believability totals** | **93** | **4.29 (0.90)** | **5 (1)** | **74** | **4.47 (0.82)** | **5 (1)** | **0.186** | **167** | **4.38 (0.86)** | **5 (1)** |
|  | | | | | | | | | | |
|  | | | | | | | | | | |
| **Category 4: Opinions on relevance, didactic content, and usefulness** | | | | | | | | | | |
| This simulation experience is an effective way to practice preoperative events � chart review, circuit selection and priming � interfacing with surgical field, cannula selection | 8 | 4.75 (0.71) | 5 (0) | 21 | 4.38 (1.12) | 5 (1) | 0.395 | 29 | 4.48 (1.02) | 5 (1) |
| The simulation experience realistically recreated: Using the simulator gave me a better understanding about CPB than I could have gained from watching a procedure in the operating room | 8 | 4.50 (0.76) | 5 (1) | 0 | \* | \* | \* | 8 | 4.50 (0.76) | 5 (1) |
| Using the simulator gave me a better understanding about CPB than watching a case | 8 | 4.75 (0.71) | 5 (0.5) | 0 | \* | \* | \* | 8 | 4.75 (0.71) | 5 (0.5) |
| Using the simulator gave me a better understanding about CPB than watching a case | 10 | 4.20 (1.48) | 5 (2) | 18 | 4.33 (1.19) | 5 (1) | 0.796 | 28 | 4.29 (1.27) | 5 (1) |
|  |  |  |  |  |  |  |  |  |  |  |
| Using the simulator gave me a better understanding about CPB than attending a lecture | 5 | 5.00 (0.00) | 5 (0) | 0 | \* | \* | \* | 5 | 5.00 (0.00) | 5 (0) |
| Using the simulator gave me a better understanding about CPB than attending a lecture | 10 | 4.50 (1.08) | 5 (1) | 18 | 4.44 (1.15) | 5 (1) | 0.901 | 28 | 4.46 (1.11) | 5 (1) |
| I clearly understood the purpose and objective of the simulation exercise | 25 | 4.56 (0.71) | 5 (1) | 18 | 4.83 (0.38) | 5 (0) | 0.242 | 43 | 4.67 (0.61) | 5 (1) |
| The background information presented today was useful to my residency | 8 | 4.62 (0.74) | 5 (1) | 0 | \* | \* | \* | 8 | 4.62 (0.74) | 5 (1) |
| The pre-briefing adequately prepared me for my simulation session | 25 | 4.32 (0.75) | 4 (1) | 18 | 4.72 (0.57) | 5 (0) | 0.083 | 43 | 4.49 (0.70) | 5 (1) |
| I was appropriately oriented to the simulator environment prior to the event | 9 | 4.11 (1.05) | 4 (2) | 20 | 4.30 (1.17) | 5 (2) | 0.683 | 29 | 4.24 (1.12) | 5 (2) |
| The debriefing provided objective and valuable feedback on my performance | 25 | 4.9 (0.28) | 5 (0) | 17 | 4.94 (0.24) | 5 (0) | 0.710 | 42 | 4.93 (0.26) | 5 (0) |
| The debriefing at the end was valuable and educational | 8 | 4.88 (0.35) | 5 (0) | 21 | 4.19 (1.25) | 5 (2) | 0.142 | 29 | 4.38 (1.11) | 5 (1) |
| **Category 4: Opinions on relevance, didactic content, and usefulness totals** | **149** | **4.57 (0.80)** | **5 (1)** | **151** | **4.50 (0.99)** | **5 (1)** | **0.483** | **300** | **4.54 (0.90)** | **5 (1)** |
|  | | | | | | | | | | |
| **All Four Categories Grand Total** | **505** | **4.44 (0.81)** | **5 (1)** | **448** | **4.35 (1.00)** | **5 (1)** | **0.24** | **953** | **4.41 (0.91)** | **5 (1)** |
|  | | | | | | | | | | |
| **Legend:** n is the number of responses to post-simulation surveys. M = Mean, SD = Standard Deviation, m = median, IQR = Interquartile Range. Between Groups p Value is ANOVA test between Experienced (> 20 human cases) versus Inexperienced means. \* Indicates no data collected. | | | | | | | | | | |
